# The Outcomes of Coronary Artery Bypass Surgery after 18 Months—Is There an Influence of the Initial Right Ventricle Diastolic Dysfunction?

**DOI:** 10.3390/jcdd10010018

**Published:** 2023-01-04

**Authors:** Alexey N. Sumin, Anna V. Shcheglova, Ekaterina V. Korok, Tatjana Ju. Sergeeva

**Affiliations:** Federal State Budgetary Scientific Institution “Research Institute for Complex Issues of Cardiovascular Disease”, Sosnoviy Blvd., 6, 650002 Kemerovo, Russia

**Keywords:** coronary artery bypass surgery, right ventricle, diastolic function, postoperative outcomes

## Abstract

Background. This study aimed to investigate the association of preoperative right heart filling indicators with outcomes after coronary artery bypass grafting (CABG) at an 18 month follow up. Methods. Patients who underwent CABG at a single center were included in this study. In addition to the baseline preoperative indicators and perioperative data, initial parameters of the right ventricle (RV) systolic and diastolic function were assessed. Results. Among the 189 patients, a total of 19 (10.0%) MACE (cardiovascular death, nonfatal myocardial infarction and stroke) were recorded during an 18 month follow up. In patients with the development of MACE during the initial examination, the following changes in RV function were revealed compared with the group without MACE: a decrease in the e’t index (8.2 versus 9.6 cm/s, *p* = 0.029), an increase in the Et/e’t ratio (5.25 vs. 4.42, *p* = 0.049) and more frequent presence of RV pseudonormal filling (*p* = 0.03). In the binary logistic regression analysis, the development of MACE 18 months after CABG was associated with the nonconduction of PCI before surgery, the presence of peripheral atherosclerosis, an increase in IVST and Et/e’t and a decrease in LVEF. Conclusions. RV diastolic dysfunction in the preoperative period was associated with the development of MACE within 18 months after CABG, and the ratio Et/e’t was one of the independent predictors of MACE in a multiple regression analysis. This makes it expedient to include an assessment of not only systolic but also diastolic RV function in the preoperative examination. The inclusion of an assessment of RV diastolic function in the pre-CABG evaluation of patients deserves further study.

## 1. Introduction

During myocardial revascularization, the state of the heart chambers, reflecting the degree of damage to the heart muscle, can determine both the tactics of treatment and the prognosis. First of all, in such cases, the state of the left ventricle is considered; for example, a decrease in the ejection fraction indicates a deterioration in the pumping function of the heart and, accordingly, is included in the preoperative risk assessment scales [1]. Other indicators of intracardiac hemodynamics (impaired filling of the left ventricle, the state of the right heart) are not used in routine clinical practice since they are not included in the risk assessment scales.

However, the assessment of the right heart in the perioperative period certainly deserves attention. So, it was shown that in the postoperative period in patients after CABG, there is a significant decrease in the function of the right ventricle. This decrease is explained both by the influence of a number of perioperative factors (sternotomy, pericardial incision and cardioplegia) and by perioperative complications (prolonged cardiopulmonary bypass, suboptimal intraoperative myocardial protection, coronary embolism with right ventricular ischemia and lung damage during mechanical ventilation) [2]. As a rule, asymptomatic dysfunction of the right ventricle (RV) does not require special treatment methods. Decreased right ventricular function after heart surgery may persist for up to a year after surgery [3,4] and even longer [5]. Clinical manifestations of right ventricular failure after cardiac surgery rarely develop, require appropriate therapy [6] and are accompanied by adverse outcomes (death, stroke, reintubation and prolonged stay in the ICU) [7]. Less severe manifestations of postoperative RV dysfunction (less than 20% reduction in systolic function with additional examination) may also be accompanied by prolonged mechanical ventilation, the need for inotropic support, an increase in creatinine and a long stay in the ICU [8]. In the longer term after surgery, a decrease in RV systolic function was associated with a decrease in the distance of 6-minute walking [4]. These data are based mainly on indicators of RV systolic dysfunction, and the perioperative dynamics of RV diastolic function is assessed much less frequently [5,9], although a recent study shows the importance of assessing and measuring the RV diastolic function of these patients [10].

Most often, the investigation of the right heart was performed before cardiac surgery in patients with a low left ventricular ejection fraction [11,12]. It has been shown that a decrease in the systolic or diastolic function of the right ventricle is associated with negative immediate results of CABG [11,12,13]. Lella et al. showed that the initial decrease in the ejection fraction of RV is associated with an increased rate of rehospitalization after heart surgery [14]. In this category of patients, the negative prognostic effect of reduced RV systolic function persisted during a 5-year follow up after CABG [11]. In patients with an unchanged preoperative left ventricular ejection fraction, RV systolic dysfunction was accompanied not only by an increased rate of perioperative complications, but also with long-term mortality after CABG [15]. It has also been shown that in the absence of obvious changes in the left ventricle before CABG surgery, initial right ventricular filling disorders were associated with the development of postoperative heart failure requiring inotropic support [10]. It is unclear whether signs of initial right ventricular diastolic dysfunction may have an impact on the prognosis of patients when assessing the midterm results of CABG, but so far no such studies have been conducted. This formed the basis for the present study, which aimed to investigate the association of preoperative right heart filling indicators with outcomes after CABG at an 18 month follow up. 

## 2. Subjects, Materials and Methods 

### 2.1. Study Population

**Figure 1:**
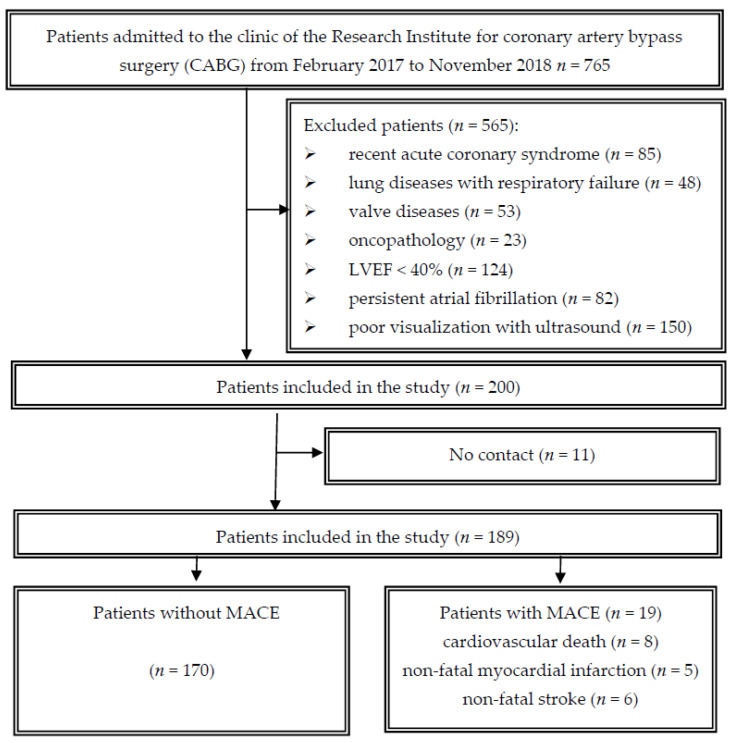
Study flowchart; MACE—major adverse cardiovascular event.

A single-center prospective cohort study was conducted at the cardiovascular surgery clinic of the Research Institute for Complex Problems of Cardiovascular Diseases. The initial cohort of 200 patients undergoing coronary bypass surgery was selected for the period from February 2017 to November 2018. The criteria for the inclusion and exclusion of patients in this study were presented in detail in previously published articles [5,10]. Subsequently, we assessed patient outcomes 18 months after surgery (Figure 1). The study protocol was approved by the Local Ethics Committee of the Research Institute for Complex Issues of Cardiovascular Diseases (Protocol No. 20170118). This study was conducted in accordance with the Helsinki Declaration. Before inclusion in the study, all patients signed an informed consent form.

### 2.2. Data Collection 

Baseline preoperative indicators were obtained from the electronic database of the CABG registry. We took into account the following indicators: demographic (gender, age, body mass index), anamnestic (old myocardial infarction, stroke, history of PCI), clinical (presence of angina pectoris, heart failure, comorbid diseases, presence of peripheral atherosclerosis) and medicine therapy. Additionally, all patients underwent a standard laboratory examination before surgery (glucose, urea, creatinine and lipid complex), ultrasound assessment of extracranial arteries and invasive coronary angiography. Among the perioperative parameters, we took into account the number of shunts, the use of a cardiopulmonary bypass, the duration of CPB, the time of aortic clamping and combined interventions (ventriculoplasty, thrombectomy, radiofrequency ablation, carotid endarterectomy and valvular surgery).

### 2.3. Echocardiographic Examination 

All patients underwent standard transthoracic echocardiography using a Philips Clear Vue 550 ultrasound scanner (USA) according to modern recommendations [16]; the study technique was presented earlier [17]. We evaluated the structural parameters of the left heart: the size and volume of the left ventricle, the mass of the LV myocardium and the diameter of the left atrium (LA). The ejection fraction of the left ventricle was assessed using the Simpson method. Among the indicators of the right heart, we determined the size of the right atrium (RA) and RV, the wall thickness of the right ventricle in diastole (RVTh) and the systolic excursion of the tricuspid ring (TAPSE).

Additionally, the diastolic function of the ventricles of the heart was assessed. In the Doppler mode, the parameters of the LV and RV in diastole were studied: the peak velocity of early (E) and late transmitral filling (A) and their ratio (E/A), isovolumetric relaxation time (IVRT), early (Et) and late (At) transtricuspid filling of the RV and their ratio (Et/At). The speed of the early and late diastolic movement of the fibrous ring of the mitral and tricuspid valves and their ratio (e’, a’, e’/a’, E/e’; e’t, a’t, e’t/a’t, Et/e’t) and the speed of the systolic movement of the mitral (s’) and tricuspid (s’t) valve rings were assessed. The Tei index for both ventricles was determined.

At values of the Et/At ratio within 0.8–2.1, the diastolic function of the right ventricle was considered normal. When the Et/At ratio values were <0.8 or >2.1, and/or the Et/et’ ratio was >6, diastolic RV dysfunction was diagnosed [18]. At the same time, values of the Et/At ratio <0.8 were a sign of the impaired relaxation of the RV, the values of Et/At in the range of 0.8–2.1 and a ratio of Et/et’ >6 were signs of the pseudonormal filling of the RV and the Et/At ratio >2.1 was a sign of a restrictive type of RV filling.

### 2.4. Follow Up

The midterm results of the CABG were assessed after 18 months during patient examination in the clinic or during telephone contact with the patient or his relatives. As adverse outcomes (MACE), the following indicators were analyzed: death, nonfatal myocardial infarction and stroke. Additionally, elective surgeries on the arteries (percutaneous coronary intervention and operations on noncoronary arteries) and any other hospitalizations for cardiovascular diseases were evaluated. For subsequent analysis, patients were divided into 2 groups: those with a favorable prognosis in prospective observation and those with the presence of events (in one variant, only MACE was taken into account, in the other, not only MACE, but also elective surgeries and any cardiovascular hospitalizations were taken into account).

### 2.5. Statistical Analyses

A statistical analysis was carried out using the standard statistical software packages “STATISTICA 8.0” (Dell Software, Inc., Round Rock, TX, USA) and SPSS 17.0 (IBM, Armonk, NY, USA). Qualitative variables were presented as absolute values and percentages, and quantitative variables were presented as medians and quartiles (25th and 75th percentiles) since the distribution differed from normal (the Shapiro–Wilk test (W-statistic) showed a significant departure from normality (*p* < 0.05) for all the analyzed variables). To compare the two groups, we used the Mann–Whitney test and the χ2 test. With a small number of observations, Fisher’s exact test was used with Yates’ correction. To identify the factors associated with the presence of unfavorable outcomes (MACE or MACE + elective surgery and cardiovascular hospitalizations) 18 months after CABG, we used a binary logistic regression (forward likelihood ratio). For the evaluation of both demographic parameters (gender and age) and baseline clinical and laboratory parameters, we included preoperative echocardiographic results and perioperative variables in the model. The performance of the variables for diagnosing the development of unfavorable outcomes (MACE or MACE + elective surgery and cardiovascular hospitalizations) was evaluated through a receiver operating characteristic curve analysis. The level of critical significance (p) during analysis was equal to 0.05.

## 3. Results

**Figure 2:**
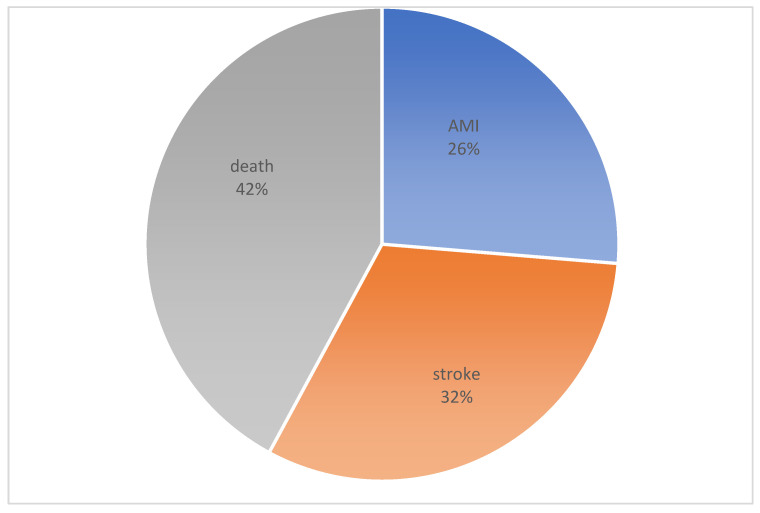
The structure of major adverse cardiovascular event after 18 months.

When evaluating the prospective follow up 18 months after CABG, it was not possible to obtain information on 11 patients. Accordingly, 189 patients were included in the analysis (Figure 1). MACE was detected in 19 patients: death in 8 patients, nonfatal myocardial infarction—in 5 patients and nonfatal stroke—in 6 patients (Figure 2). In a further analysis, we compared the initial preoperative and perioperative parameters in groups with a favorable outcome (without MACE, *n* = 170) and with an unfavorable outcome (with MACE, *n* = 19).

### AMI—Acute Myocardial Infarction

Additionally, we evaluated the frequency of elective interventions on the arteries and emergency hospitalization during the observation period: PCI was performed in two cases, operations on the noncoronary arteries were performed in six cases, unstable angina without myocardial infarction was detected in ten patients and rhythm disturbances were detected in six cases. Based on the data obtained, groups were formed with a favorable outcome (*n* = 146) and an unfavorable outcome (*n* = 43).

Table 1 presents the baseline clinical characteristics in the groups with a favorable outcome and with the development of MACE. As can be seen, there were no significant differences between the groups in most of the initial parameters. Significantly more often in the MACE group there were patients with peripheral atherosclerosis (*p* = 0.038) and with symptoms of chronic heart failure in at least the III functional class (*p* = 0.014). Additionally, some of the differences had borderline statistical significance: in the group with MACE, there were slightly more men (*p* = 0.052), more often in the anamnesis of myocardial infarction (*p* = 0.053), and the duration of CPB was somewhat longer (*p* = 0.053) (Table 1).

The initial dimensions and volumes of the left ventricle in the MACE group were higher than in the group without MACE (Table 2): the median EDD was 6.0 cm and 5.5 cm, respectively (*p* = 0.027); ESD—4.1 cm and 3.6 cm, *p* = 0.005; EDV—180.0 mL and 147.0 mL, *p* = 0.028; and ESV—70.0 mL and 51.0 mL, *p* = 0.008. These differences can be explained by the greater predominance of men in the MACE group and the higher incidence of old myocardial infarction; they were also accompanied by a decrease in EF (56.0 vs. 61.0, respectively, *p* = 0.005) and an increase in ESVi (35.2 vs. 27.28, *p* = 0.025) in this group. At the same time, there were no differences between the groups in parameters of the LV diastolic function.

The initial indicators of the systolic function of the right ventricle in the groups did not differ (Table 3). Additionally, there were no differences in the frequency of detection and severity of diastolic RV dysfunction in the groups. However, individual indicators of the diastolic function of the right ventricle in the groups differed: in the MACE group, the *e*’t index was lower (8.2 versus 9.6 cm/s, *p* = 0.029) and the Et/e’t ratio was higher (5.25 vs. 4.42, *p* = 0.049). As a result, although the overall incidence of RV diastolic dysfunction did not differ in the groups, pseudonormal filling was significantly more often detected in the MACE group (*p* = 0.03). These data indicate more pronounced initial disorders of the RV filling in the group with the presence of MACE.

When comparing groups with an unfavorable outcome (MACE + cardiovascular hospitalizations) and with a favorable outcome (Supplementary Tables S1–S3), the differences between the groups were less pronounced. In the patients with unfavorable outcomes, rhythm disturbances occurred more often before surgery (*p* = 0.019), they took aspirin less often (*p* = 0.011) and had higher values of the LV wall thickness. Accordingly, signs of LV diastolic dysfunction were more pronounced in this group than in patients with favorable outcomes (median e’ 8.4 cm/s versus 9.4 cm/s, *p* = 0.007, e’/a’ ratio 0.75 and 0.92, respectively, *p* = 0.05). Initial indicators of the right heart in the groups with a favorable and unfavorable outcome did not differ.

When conducting a binary logistic regression analysis, the development of MACE 18 months after CABG was associated with the nonconduction of PCI before surgery, the presence of peripheral atherosclerosis, an increase in IVST and Et/e’t and a decrease in LVEF (Table 4). The logistic regression model was statistically significant, χ2(4) = 28.104, *p* = 0.001. The model explained 29.9% (Nagelkerke R^2^) of the MACE developmental variance and correctly classified 89.7% of cases. An unfavorable outcome 18 months after CABG was significantly (χ2(2) = 28.18, *p* = 0.005) associated with the presence of III FC according to NYHA, the presence of arrhythmia, an increase in IVST and not taking aspirin (Table 5). The model explained 22.4% (Nagelkerke R^2^) of the MACE developmental variance and correctly classified 80.3% of cases.

**Figure 3:**
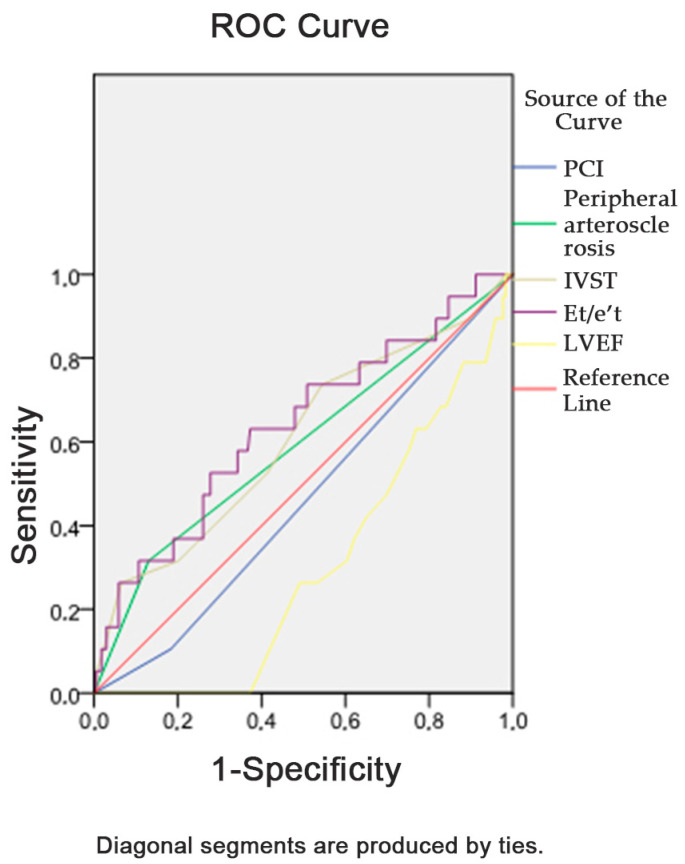
Receiver operating characteristic curve analysis. Performance of preoperative parameters in discriminating the risk of MACE development 18 months after CABG. Notes: ROC, receiver operating characteristic; Et/e’t, ratio of early transtricuspid diastolic filling to the early diastolic tricuspid annular tissue velocity; LVEF, left ventricular ejection fraction; IVST, thickness of the interventricular septum; PCI, percutaneous coronary intervention; TAPSE, tricuspid annular plane systolic excursion.

The association of the initial parameters with the development of MACE is presented in Figure 3. As shown in Supplementary Table S4, the areas under the curves were maximal for the increased Et/e’t (0.638) and decreased LVEF (0.697). However, these and the rest of the parameters (peripheral atherosclerosis, PCI and IVST) were <0.7, indicating an unacceptable ability to distinguish. The association of the initial parameters with the development of the unfavorable outcome is presented in Supplementary Figure S1 and Supplementary Table S5. The areas under the curves were maximal for the increased IVST (0.631). 

## 4. Discussion

The present study assessed the possible prognostic value of right ventricular diastolic dysfunction during a follow up of patients after CABG. We were able to show that in addition to the clinical parameters (the presence of peripheral atherosclerosis and no history of PCI) and echocardiographic parameters of the left ventricle (IVST and LVEF), the parameters of the RV filling (Et/e’t ratio) had an independent effect on the development of MACE within 18 months after CABG.

It has previously been shown that RV systolic dysfunction before cardiac surgery is associated with poor prognosis. Thus, in a multicenter prospective study involving more than 900 patients, it was shown that the preoperative assessment of systolic function and RV dimensions can significantly improve the prediction of in-hospital mortality during heart surgery compared to the isolated evaluation Euroscore II scale (an increase in the area under the curve from 0.86 to 0.96, *p* = 0.045) [19]. In valvular heart surgery, preoperative RV dysfunction was associated with a 3.5-fold increased risk of postoperative 30-day mortality and a 4.2-fold increased risk of multiple postoperative adverse events. In this study, we also used a comprehensive assessment of RV function based primarily on indicators of its systolic function [20]. Moreover, the initial systolic RV dysfunction led to increased mortality (both total and cardiovascular) during a three-year follow up of patients after heart surgery [21]. With isolated CABG, similar results were obtained—preoperative RV systolic dysfunction was accompanied by both an increase in the number of postoperative adverse outcomes and a worsening of patient survival during a follow up 3.5 years after the surgery [15]. Additionally, in a prospective follow up after CABG in patients with a low LVEF, a low baseline systolic RV function was one of the independent predictors of cardiovascular mortality for 4.5 years (hazard ratio 2.14; *p* = 0.034) [11]. The above studies are united by two circumstances—they included patients at an initially increased risk (valvular surgery and an initial low LV ejection fraction), and an assessment of the RV systolic function was used in prognostic models. As shown earlier, in patients with a less severe pathology, systolic RV dysfunction is rare, in contrast to diastolic RV dysfunction [10]. Therefore, it would be interesting to compare the effect of the diastolic function of the RV on the results of cardiac surgery.

There have been few such studies to date. Thus, in a cohort of patients with low LVEF (<35%) undergoing CABG, a significant increase in preoperative Et/e’t ≥10 and the absence of suitable target bypass arteries were significantly associated with 30-day postoperative mortality [12]. In a recent study assessing the short-term prognosis after CABG in patients with moderate–severe LV systolic dysfunction, an increase in the Et/e’t ratio was accompanied by an increase in the incidence of postoperative atrial fibrillation but did not affect mortality within 2 months after surgery [22]. In patients without initial systolic dysfunction, the presence of preoperative diastolic RV dysfunction was accompanied by a more frequent development of postoperative heart failure after CABG (odds ratio 4.82; *p* = 0.015) [10]. Our study shows for the first time that RV diastolic dysfunction is associated not only with short-term but also medium-term prognosis after CABG in patients without initial left ventricular systolic dysfunction. It should also be noted that among the indicators of RV diastolic dysfunction studied by us, the ratio Et/e’t was the most informative, as in the above studies [12,22].

Currently, the standard perioperative risk assessment for cardiac surgery does not include preoperative measures of right ventricular function. This is explained primarily by the fact that the routine assessment of RV function is not included in the scope of the preoperative examination. The studies of recent years that we cited above and the reviews [23,24] emphasize the need to assess the RV systolic function before cardiac surgery for a more accurate assessment of the prognosis. The present study emphasizes the need for a more comprehensive assessment of the RV function, with a study of the indicators of RV filling as well.

### Study Limitations

When evaluating the results of this study, a number of its limitations should be taken into account. First, this study was conducted in a single center, so the applicability of its results to other patient cohorts has not been proven. Secondly, we took into account only preoperative echocardiographic parameters, but did not take into account their changes immediately after surgery, although it is a decrease in RV function that is more often detected in the postoperative period. To overcome this limitation, we took into account perioperative indicators that could affect the deterioration of RV function in our binary logistic regression model; they did not affect the incidence of MACE. Thirdly, we did not use some modern methods for evaluating RV function (speckle tracking and 3D echocardiography). As clinical practice shows, they are still rarely used [25]; in addition, we were able to show the value of assessing the RV diastolic function conventional methods. Next, in this study, we did not include patients with comorbid conditions or with the presence of concomitant valvular lesions, so its results are difficult to generalize to the entire cohort of patients before CABG. Finally, in the present study, we did not perform an in-depth analysis of the results from a hemodynamic perspective. Indeed, the low flow rate in the bypass graft could potentially lead to a higher risk of atherosclerotic plaque growth and restenosis [26]. In addition, the stiffness and geometry of the shunt, as well as the rheology in it [27], can significantly affect focal hemodynamics and the risk of restenosis. However, the design of this study did not include an assessment of the state of shunts and coronary arteries in dynamics.

## 5. Conclusions

Our study showed that the development of MACE (death, nonfatal myocardial infarction and nonfatal stroke) within 18 months after CABG was associated with such independent factors as the presence of peripheral atherosclerosis, a history of non-PCI, an increase in the thickness of the interventricular septum, a decrease in LVEF and an increase in the ratio Et/e’t. The latter indicator characterizes the RV diastolic dysfunction in the preoperative period. This makes it expedient to include an assessment of not only the systolic but also diastolic RV function in the preoperative examination.

## Figures and Tables

**Table 1:** Demographic data and clinical characteristics of patients with and without MACE 18 months after CABG.

Characteristics	Group 1 without MACE (*n* = 170)	Group 2 MACE (*n* = 19)	*p*
Clinical and anamnestic data			
Male, *n* (%)	127 (74.71)	18 (94.74)	0.05
Age, ME (LQ, UQ) (y)	64.0 (60.0;68.0)	64.0 (62.0;67.0)	0.613
Body mass index, ME (LQ, UQ) (kg/m^2^)	28.7 (25.7;30.5)	29.4 (27.3;33.3)	0.129
Smoking, *n* (%)	50 (30.12)	7 (36.84)	0.547
Hypertension, *n* (%)	18 (94.74)	161 (94.71)	0.995
Angina pectoris, *n* (%)	147 (86.47)	18 (94.74)	0.304
Myocardial infarction history, *n* (%)	105 (61.76)	16 (84.21)	0.053
Rhythm disturbances, *n* (%)	31 (18.34)	4 (21.05)	0.773
Diabetes mellitus, *n* (%)	43 (25.29)	7 (36.84)	0.279
Stroke history, *n* (%)	15 (8.82)	2 (10.53)	0.805
PCI history, *n* (%)	32 (18.82)	2 (10.53)	0.371
CHF NYHA III FC, *n* (%)	31 (18.24)	8 (42.11)	0.014
Carotid atherosclerosis >50%, *n* (%)	33 (19.41)	3 (15.76)	0.702
Hyperlipidemia, *n* (%)	113 (66.47)	10 (52.63)	0.231
Therapy before surgery			
Betablocker, *n* (%)	157 (62.35)	17 (89.47)	0.659
Statins, *n* (%)	163 (95.88)	18 (94.74)	0.814
Calcium-channel blockers, *n* (%)	122 (71.76)	16 (84.21)	0.246
ACE-I, *n* (%)	130 (76.47)	15 (78.95)	0.808
Aspirin, *n* (%)	158 (92.94)	17 (89.47)	0.584
Surgical procedure			
Ventriculoplasty, *n* (%)	14 (8.24)	2 (10.53)	0.733
Thrombectomy, *n* (%)	8 (4.71)	1 (5.26)	0.913
Radiofrequency ablation, *n* (%)	5 (2.94)	0	0.448
Carotid endarterectomy, *n* (%)	18 (10.59)	3 (15.79)	0.493
Bypass graft number, ME (LQ, UQ)	3.0 (2.0;3.0)	3.0 (2.0;3.0)	0.089
Cardiopulmonary bypass duration, ME (LQ; UQ) (min)	76.0 (65.0;92.0)	89.5 (74.0;99.0)	0.053
Aortic cross-clamp time, ME (LQ; UQ) (min)	50.0 (40.0;61.0)	59.5 (51.0;64.0)	0.092

Continuous data are presented as the median (lower quartile, upper quartile). CHF, chronic heart failure; NYHA, New York Heart Association; FC, functional class; PCI, percutaneous coronary intervention; ACE-I, angiotensin-converting enzyme inhibitor.

**Table 2:** LV indicators before surgery in patients with and without MACE 18 months after CABG.

Characteristics	Group 1 without MACE (*n* = 170)	Group 2 MACE (*n* = 19)	*p*
Structural indicators and systolic function			
Aorta, mm ME (LQ, UQ)	3.5 (3.3;3.8)	3.5 (3.4;3.8)	0.547
LA, ME (LQ; UQ)mm	4.4 (4.1;4.8)	4.6 (4.3;5.0)	0.168
EDD, ME (LQ; UQ) mm	5.5 (5.2;6.1)	6.0 (5.5;6.2)	0.027
EDDi, ME (LQ; UQ) mm/m^2^	2.91 (2.76;3.16)	2.95 (2.76;3.01)	0.94
ESD, ME (LQ;UQ) mm	3.6 (3.3;4.0)	4.1 (3.8;4.3)	0.005
ESDi, ME (LQ; UQ) mm/m^2^	1.87(1.71;2.11)	2.02(1.89;2.2)	0.14
EDV, ME (LQ;UQ) mL	147.0 (130.0;187.0)	180.0 (147.0;194.0)	0.028
EDVi, ME (LQ;UQ) mL/m^2^	78.9(70.33;96.5)	87.67(81.17;93.14)	0.126
ESV, ME (LQ;UQ) mL	51.0 (43.0;70.0)	70.0 (51.0;83.0)	0.008
ESVi, ME (LQ;UQ) mL/m^2^	27.28(21.9;35.1)	35.2(25.03;43.06)	0.025
LVEF, ME (LQ;UQ) %	61.0 (55.0;66.0)	56.0 (50.0;62.0)	0.005
SV, ME (LQ, UQ), mL	147.0 (130.0;187.0)	177.0 (150.5;194.0)	0.271
LVM, ME (LQ;UQ) (g)	304.3 (250.4;364.7)	315.3 (288.7;428.8)	0.243
LVMi, ME (LQ;UQ)	152.5 (128.9;184.0)	177.8 (151.3;243.5)	0.172
IVST ME (LQ, UQ) cm	1.1 (1.0;1.2)	1.2 (1.0;1.4)	0.128
PW LV, ME (LQ, UQ) cm	1.1 (1.0;1.2)	1.1 (1.0;1.3)	0.361
Indicators of LV diastolic function			
IVRT, ME(LQ;UQ) m/s	92.0 (90.0;98.0)	94.0 (92.0;99.0)	0.155
E, cm/sME (LQ, UQ)	59.0 (45.0;68.0)	52.0 (42.0;63.0)	0.082
A, cm/s ME (LQ, UQ)	67.0 (57.0;79.0)	72.0 (63.0;77.0)	0.667
E/A	0.78 (0.66;1.13)	0.76 (0.63;0.82)	0.131
e’, cm/s, ME (LQ, UQ)	9.0 (7.5;11.0)	8.4 (7.5;11.9)	0.529
a’, cm/sME (LQ, UQ)	9.8 (8.4;11.6)	10.8 (9.8;11.4)	0.385
e’/a’, ME (LQ, UQ)	0.9 (0.68;1.29)	0.73 (0.66;1.2)	0.264
s’, cm/s ME (LQ, UQ)	9.0 (8.0;10.2)	9.6 (8.4;11.6)	0.308
E/e’, ME (LQ, UQ)	6.14 (4.8;7.67)	5.56 (4.62;6.91)	0.165
Tei LV (LQ;UQ)	0.32 (0.25;0.42)	0.33 (0.26;0.41)	0.954

Continuous data are presented as the median (lower quartile, upper quartile): EDD—end-diastolic dimension; EDDi—end-diastolic dimension index; EDV—end-diastolic volume; EDVi—end-diastolic volume index; EF—ejection fraction; LA—left atrium; LVMi—left ventricular mass index; ESV—end-systolic volume; ESVi—end-systolic volume index; ESD—end-systolic dimension; ESDi—end-systolic dimension index; IVRT—isovolumic relaxation time; IVST—interventricular septum thickness; PW LV—posterior wall of the left ventricle; E—peak early diastolic left ventricular filling velocity; A—peak left ventricular filling velocity at atrial contraction; E/A—ratio of peak early diastolic filling velocity to peak filling velocity at atrial contract; e’—early diastolic mitral annular tissue velocity; a’—late diastolic mitral annular tissue velocity; e’/a’—ratio of the velocities of early and late movements of the mitral annulus; s’—systolic mitral annular tissue velocity; E/e’—ratio of the early diastolic velocity of mitral inflow to the early diastolic velocity of mitral annular motion; Tei LV—myocardial performance index left ventricular.

**Table 3:** Indicators of the right ventricle before surgery in patients with and without MACE 18 months after CABG.

Characteristics	Group 1 without MACE (*n* = 170)	Group 2 MACE (*n* = 19)	*p*
Structural indicators and systolic function			
RV, (LQ; UQ) mm	2.0 (1.8;2.2)	2.1 (2.0;2.4)	0.063
RVth, (LQ; UQ) mm	0.4 (0.3;0.4)	0.4 (0.3;0.4)	0.714
TAPSE, (LQ, UQ) mm	23.0 (20.0;26.0)	24.0 (22.0;28.0)	0.261
RVEF, ME (LQ;UQ) %	55.0 (52.0;57.0)	54.0 (53.0;56.0)	0.682
RA, ME (LQ; UQ) mm	40.0 (32.0;49.0)	43.0 (39.0;52.0)	0.175
mPAP. (LQ; UQ) mmhg	12.0 (11.0;13.0)	15.0 (12.0;20.0)	0.057
sPAP. (LQ; UQ) mmhg	25.0 (24.0;29.0)	28.0 (25.0;30.0)	0.158
Indicators of RV diastolic function			
E_t_, ME (LQ, UQ) cm/s	44.0 (37.0;49.0)	44.0 (37.0;50.0)	0.836
A_t_, ME (LQ, UQ) cm/s	42.0 (34.0;49.5)	41.0 (37.0;48.0)	0.759
E_t_/A_t_	1.1 (0.76;1.36)	0.88 (0.78;1.35)	0.934
e’_t_, ME (LQ, UQ) cm/s	9.6 (8.4;11.3)	8.2 (7.3;10.2)	0.029
a’_t_, ME (LQ, UQ) cm/s	14.0 (12.1;16.1)	13.0 (10.8;15.6)	0.239
e’_t_/a’_t_, ME (LQ, UQ)	069 (0.59;0.79)	0.68 (0.57;0.77)	0.326
s’_t_, ME (LQ, UQ) cm/s	13.1 (11.9;14.8)	13.3 (11.8;15.0)	0.926
E_t_/e’_t_, ME (LQ, UQ)	4.42 (3.6;5.5)	5.25 (3.98;6.85)	0.049
RV Tei index (LQ; UQ)	0.3 (0.23;0.38)	0.3 (0.2;0.36)	0.566
RVDD, *n* (%)	73 (42.9)	10 (52.6)	0.449
- impaired relaxation, *n* (%)	51 (30.0)	4 (21.1)	0.416
- pseudonormal filling, *n* (%)	22 (12.9)	6 (31.6)	0.030

Continuous data are presented as the median (lower quartile, upper quartile). RV—right ventricular; mPAP—mean pulmonary arterial pressure; sPAP—systolic pulmonary arterial pressure; TAPSE—tricuspid annular plane systolic excursion; Tei—myocardial performance index; RVth—thickness of right ventricular wall in diastole; EF—ejection fraction; RA—right atrium; Et—early transtricuspid diastolic filling; At—late transtricuspid mitral diastolic filling; e’t—early diastolic tricuspid annular tissue velocity; a’t—late diastolic tricuspid annular tissue velocity; e’t/a’t—ratio of early diastolic tricuspid annular tissue velocity to the late diastolic tricuspid annular tissue velocity; s’t—systolic tricuspid annular tissue velocity; Et/e’t—ratio of early transmitral diastolic filling to the early diastolic tricuspid annular tissue velocity; RVDD—right ventricular diastolic dysfunction; RVSD—right ventricular systolic dysfunction.

**Table 4:** Association of variables before surgery with MACE 18 months after CABG (binary logistic regression analysis, forward likelihood ratio).

	B	S.E.	Wald	df	Sig.	Exp(B)
PCI	−1.937	0.957	4.100	1	0.043	0.144
PA	1.770	0.670	6.965	1	0.008	5.868
IVST	4.338	1.654	6.879	1	0.009	76.567
Et/e’t	0.408	0.173	5.551	1	0.018	1.504
LVEF	−0.129	0.037	12.098	1	0.001	0.879
Constant	−1.827	2.342	0.609	1	0.435	0.161

PCI, percutaneous coronary intervention; PA, peripheral atherosclerosis; IVST, interventricular septum thickness; Et/e’t, ratio of early transtricuspid diastolic filling to the early diastolic tricuspid annular tissue velocity; LVEF, left ventricular ejection fraction.

**Table 5:** Association of variables before surgery with unfavorable outcome (MACE + cardiovascular hospitalization) 18 months after CABG (binary logistic regression analysis, forward likelihood ratio).

	B	S.E.	Wald	df	Sig.	Exp(B)
NYHA III FC	1.298	0.451	8.278	1	0.004	3.661
Arrhythmia	0.905	0.454	3.965	1	0.046	2.471
Aspirin	−1.973	0.647	9.302	1	0.002	0.139
IVST	3.444	1.190	8.373	1	0.004	31.308
Constant	−3.805	1.413	7.248	1	0.007	0.022

NYHA, New York Heart Association; FC, functional class; IVST, interventricular septum thickness.

## Data Availability

The datasets used and/or analyzed during the current study are available from the corresponding author upon reasonable request.

## Supplementary Materials

The following supporting information can be downloaded at: https://www.mdpi.com/article/10.3390/jcdd10010018/s1, Figure S1: Receiver operating characteristic curve analysis. Performance of preoperative parameters in dis-criminating the risk of MACE development 18 months after CABG. Notes: ROC, receiver operating characteristic; IVST, thickness of the interventricular septum; NYHA, New York Heart Association; FC, functional class; Table S1: Demographic data and clinical characteristics of patients with and without unfavorable outcome 18 months after CABG; Table S2: LV indicators before surgery in patients with and without unfavorable outcome 18 months after CABG; Table S3: Indicators of the right ventricle before sugery in patients with and without unfavorable outcome 18 months after CABG; Table S4: Area Under the Receiver Operating Characteristic Curve (Performance of Preoperative Parameters in Discriminating the Risk of MACE Development 18 months after CABG); Table S5: Area Under the Receiver Operating Characteristic Curve (Performance of Preoperative Parameters in Discriminating the Risk of unfavorable outcome (MACE + cardiovascular hospitalization) 18 months after CABG).
