# Supplementary material for: The Outcomes of Coronary Artery Bypass Surgery after 18 Months—Is There an Influence of the Initial Right Ventricle Diastolic Dysfunction?
Source: J Cardiovasc Dev Dis. 2023 Jan 4;10(1):18. doi: 10.3390/jcdd10010018 (PMC9866549; doi:10.3390/jcdd10010018)
Supplement: Supplementary file 1 [file jcdd-10-00018-s001.zip › jcdd-2097704-supplementary.pdf]

Journal of
Cardiovascular Development and Disease

Supplementals

The Outcomes of Coronary Artery Bypass Surgery after 18 Months—Is There an Influence of the Initial Right Ventricle Diastolic Dysfunction?

Alexey N. Sumin *, Anna V. Shcheglova, Ekaterina V. Korok and Tatjana Ju. Sergeeva

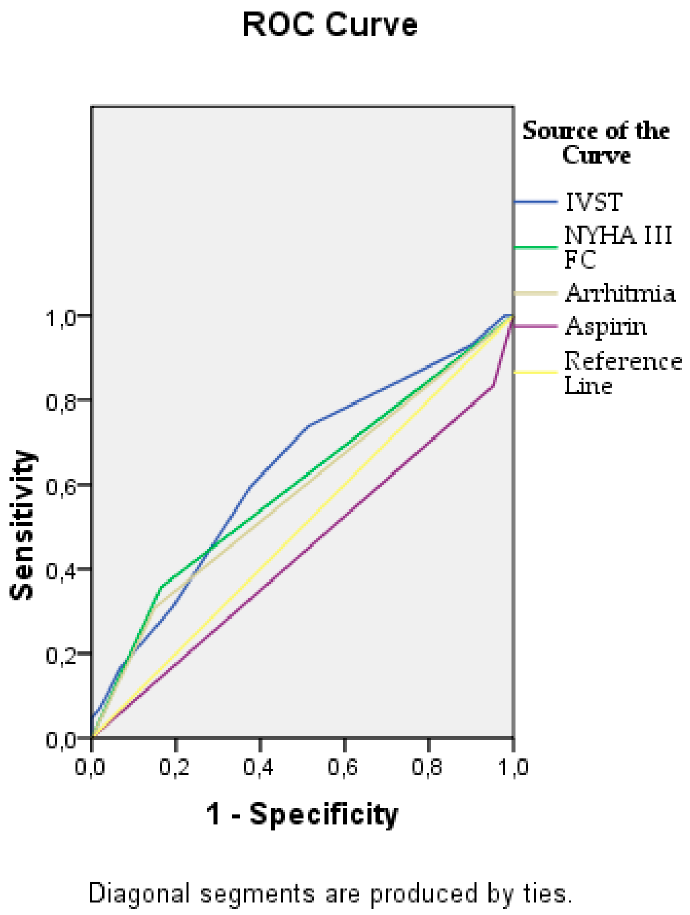

Figure S1. Receiver operating characteristic curve analysis. Performance of preoperative parameters in discriminating the risk of MACE development 18 months after CABG. Notes: ROC, receiver operating characteristic; IVST, thickness of the interventricular septum; NYHA, New York Heart Association; FC, functional class.

Table S1. Demographic data and clinical characteristics of patients with and without unfavorable outcome 18 months after CABG.

Characteristics	Group 1 Without unfavorable outcome (n = 146)	Group 2 With unfavorable outcome (n = 43)	p
Clinical and anamnestic data			

Male, n (%)	111 (76.03)	34 (79.07)	0.678
Age, ME [LQ, UQ] (y)	64.0 [60.0;68.0]	64.0 [62.0;69.0]	0.361
Body mass index, ME [LQ, UQ] (kg/m2)	28.54 [25.6;30.78]	28.71 [26.7;31.2]	0.768
Smoking, n (%)	42 (29.58)	15 (34.88)	0.509
Hypertension, n (%)	138 (94.52)	41 (95.35)	0.831
Angina pectoris, n (%)	129 (88.36)	36 (83.72)	0.422
Myocardial infarction history, n (%)	93 (63.7)	28 (65.12)	0.864
Rhythm disturbances, n (%)	22 (15.07)	13 (30.2)	0.019
Diabetes mellitus, n (%)	34 (23.29)	16 (37.21)	0.068
Stroke history, n (%)	11 (7.53)	6 (13.95)	0.195
PCI history, n (%)	27 (18.49)	7 (16.28)	0.739
CHF NYHA III FC, n (%)	24 (16.44)	15 (34.88)	0.008
Carotid atherosclerosis >50%, n (%)	28 19.18)	8 (18.6)	0.932
Hyperlipidemia, n (%)	95 65.07)	28 (65.12)	0.995
Therapy before surgery			
Betablocker, n (%)	38 (88.37)	136 (93.15)	0.308
Statins, n (%)	40 (93.02)	141 (96.58)	0.309
Calcium-channel blockers, n (%)	36 (83.72)	102 (69.86)	0.071
ACE-I, n (%)	33 (76.74)	112 (76.71)	0.995
Aspirin, n (%)	36 (83.72)	139 (95.21)	0.011
Surgical procedure			
Ventriculoplasty, n (%)	13 (8.9)	3 (6.98)	0.689
Thrombectomy, n (%)	8 (4.48)	1 (2.33)	0.393
Radiofrequency ablation, n (%)	4 2.74)	1 (2.33)	0.881
Carotid endarterectomy, n (%)	15 (10.27)	6 (13.95)	0.499
Bypass graft number, ME [LQ, UQ]	3 [2.0;3.0]	3 [2.0;3.0]	0.327
Cardiopulmonary bypass duration, ME [LQ; UQ] (min)	76.0 [65.0;90.0]	82.0 [70.0;98.0]	0.199
Aortic cross-clamp time, ME [LQ; UQ] (min)	50.0 [40.0;62.0]	53.0 [41.0;61.0]	0.674

CHF, chronic heart failure; NYHA, New York Heart Association; FC, functional class; PCI, percutaneous coronary intervention; ACE-I, angiotensin-converting enzyme inhibitor.

Table S2. LV indicators before surgery in patients with and without unfavorable outcome 18 months after CABG.

**Characteristics**	**Group 1 Without adverse outcome (*n* = 146)**	**Group 2 With adverse outcome (*n* = 43)**	**p**
Structural indicators and systolic function			
Aorta, mm ME [LQ, UQ]	3.5 [3.3;3.8]	3.6 [3.4;3.8]	0.618
LA, ME [LQ; UQ]mm	4.4 [4.1;4.7]	4.6 [4.2;5.0]	0.143
EDD, ME [LQ; UQ] mm	5.5 [5.2;6.1]	5.6 [5.3;6.1]	0.469
ESD LV, ME [LQ;UQ] mm	3.6 [3.3;4.0]	3.8 [3.4;4.2]	0.304
EDV, ME [LQ;UQ] mL	147.0 [130.0;187.0]	154.0 [135.0;187.0]	0.156
ESV, ME [LQ;UQ] mL	51.0 [42.0;70.0]	62.0 [47.0;78.0]	0.479
LVEF, ME [LQ;UQ] %	61.0 [54.0;66.0]	60.0 [55.0;65.0]	0.388
SV, ME [LQ, UQ], mL	160.0 [135.0;190.5]	147.0 [124.0;187.0]	0.301
LVM, ME [LQ;UQ] (g)	302.9 [242.1;357.2]	328.5 [276.3;391.1]	0.121

LVMI, ME [LQ;UQ]	146.8 [128.0;180.4]	177.4 [143.6;197.7]	0.132
IVST ME [LQ, UQ] cm	1.1 [1.0;1.2]	1.2 [1.0;1.3]	0.008
PW LV, ME [LQ, UQ] cm	1.0 [1.0;1.2]	1.2 [1.0;1.3]	0.023
Indicators of LV diastolic function			
IVRT, ME[LQ;UQ] m/s	92.0 [90.0;98.0]	92.0 [90.0;98.0]	0.176
E, cm/sME [LQ, UQ]	58.0 [46.0;68.0]	56.0 [44.0;66.0]	0.458
A, cm/s ME [LQ, UQ]	66.5 [57.0;78.0]	71.0 [63.0;78.0]	0.212
E/A	0.8 [0.66;1.17]	0.76 [0.66;0.87]	0.152
e', cm/s, ME [LQ, UQ]	9.4 [8.0;11.0]	8.4 [7.1;10.6]	0.007
a', cm/sME [LQ, UQ]	9.8 [8.3;11.6]	10.6 [9.2;11.6]	0.216
e'/a', ME [LQ, UQ]	0.92 [0.72;1.29]	0.75 [0.66;1.14]	0.05
s', cm/s ME [LQ, UQ]	9.2 [8.2;10.2]	8.8 [7.7;10.6]	0.619
E/e', ME [LQ, UQ]	6.0 [4.75;7.56]	6.39 [5.28;7.6]	0.595
Tei LV [LQ;UQ]	0.33 [0.25;0.43]	0.32 [0.25;0.39]	0.493

Continuous data are presented as the median [lower quartile, upper quartile]. EDD—end-diastolic dimension; EDVI—end-diastolic volume index; EF— ejection fraction; LA—left atrium; LVMI—left ventricular mass index; ESV—end-systolic volume; ESV— end-diastolic volume ESD—end-systolic dimension; IVRT—isovolumic relaxation time; IVST—interventricular septum thickness; PW LV—posterior wall of the left ventricle;E—peak early diastolic left ventricular filling velocity; A—peak left ventricular filling velocity at atrial contraction; E/A—ratio of peak early diastolic filling velocity to peak filling velocity at atrial contract; e'—early diastolic mitral annular tissue velocity; a'—late diastolic mitral annular tissue velocity; e'/a'—ratio of the velocities of early and late movements of the mitral annulus; s'—systolic mitral annular tissue velocity; E/e'— ratio of the early diastolic velocity of mitral inflow to the early diastolic velocity of mitral annular motion; Tei LV—myocardial performance index left ventricular.

Table S3. Indicators of the right ventricle before sugery in patients with and without unfavorable outcome 18 months after CABG.

**Characteristics**	**Group 1 Without adverse outcome (*n* = 146)**	**Group 2 With adverse outcome (*n* = 43)**	**p**
Structural indicators and systolic function			
RV, [LQ; UQ] mm	2.0 [1.9;2.2]	2.1 [1.8;2.3]	0.348
RVth, [LQ; UQ] mm	0.4 [0.3;0.4]	0.4 [0.3;0.4]	0.266
TAPSE, [LQ, UQ] mm	23.0 [20.0;26.0]	22.0 [21.0;26.0]	0.851
RVEF, ME [LQ;UQ] %	55.0 [52.0;57.0]	55.0 [53.0;57.0]	0.577
RA, ME [LQ; UQ] mm	40.0 [32.0;49.0]	40.0 [34.0;50.0]	0.721
mPAP. [LQ; UQ] mmhg	12.0 [11.0;13.0]	12.0 [11.0;16.0]	0.506
sPAP. [LQ; UQ] mmhg	25.0 [23.5;28.0]	28.0 [25.0;30.0]	0.051
Indicators of diastolic function			
E_t, ME [LQ, UQ] cm/s	45.0 [37.5;50.]	41.0 [36.0;49.0]	0.205
A_t, ME [LQ, UQ] cm/s	42.0 [34.0;49.0]	43.0 [37.0;50.0]	0.383
E_t/A_t	1.13 [0.78;1.38]	0.88 [0.74;1.28]	0.173
e'_t, ME [LQ, UQ] cm/s	9.5 [8.3;11.2]	9.4 [8.0;11.6]	0.822
a'_t, ME [LQ, UQ] cm/s	14.0 [12.1;15.8]	14.0 [12.0;16.8]	0.947
e'_t/a'_t, ME [LQ, UQ]	0.69 [0.59;0.78]	0.71 [0.58;0.81]	0.772
s'_t, ME [LQ, UQ] cm/s	13.2 [11.85;14.9]	13.0 [11.9;14.3]	0.649
E_t/e'_t, ME [LQ, UQ]	4.54 [3.65;5.58]	4.4 [3.1;5.33]	0.505
RV Tei index [LQ; UQ]	0.3 [0.22;0.38]	0.3 [0.24;0.37]	0.983
RVDD, n (%)	61 (41.8)	22 (51.2)	0.353

- impaired relaxation, n (%)	40 (27.4)	15 (34.9)	0.342
- pseudonormal filling, n (%)	21 (14.4)	7 (16.3)	0.758

Continuous data are presented as the median [lower quartile, upper quartile]. RV—right ventricular; mPAP—mean pulmonary arterial pressure; sPAP— systolic pulmonary arterial pressure; TAPSE—tricuspid annular plane systolic excursion; Tei—myocardial performance index; RVth—thickness of right ventricular wall in diastole; EF—ejection fraction; RA—right atrium; Et—early transtricuspid diastolic filling; At—late transtricuspid mitral diastolic filling; e't—early diastolic tricuspid annular tissue velocity; a't—late diastolic tricuspid annular tissue velocity; e't/a't—ratio of early diastolic tricuspid annular tissue velocity to the late diastolic tricuspid annular tissue velocity; s't—systolic tricuspid annular tissue velocity; Et/e't—ratio of early transtricuspid diastolic filling to the early diastolic tricuspid annular tissue velocity; RVDD —right ventricular diastolic dysfunction; RVSD—right ventricular systolic dysfunction.

Table S4. Area Under the Receiver Operating Characteristic Curve (Performance of Preoperative Parameters in Discriminating the Risk of MACE Development 18 months after CABG).

Test Result Variable(s)	Area	Std. Error[a]	Asymptotic Sig.[b]	Asymptotic 95% Confidence Interval	
				Lower Bound	Upper Bound
**PCI**	**0.461**	**0.067**	0.577	0.330	0.592
Peripheral atherosclerosis	0.593	0.074	0.185	0.447	0.739
IVST	0.606	0.073	0.129	0.464	0.749
Et/*e*'t	0.638	0.070	0.048	0.501	0.776
LVEF	0.303	0.052	0.005	0.202	0.404

The test result variable(s): PCI, Peripheral arterosclerosis, IVS, Et/e't, LVEF has at least one tie between the positive actual state group and the negative actual state group; a. Under the nonparametric assumption; b. Null hypothesis: true area = 0.5; PCI, percutaneous coronary intervention; IVST, interventricular septum thickness; Et/e't, ratio of early transtricuspid diastolic filling to the early diastolic tricuspid annular tissue velocity; LVEF, left ventricular ejection fraction.

Table S5. Area Under the Receiver Operating Characteristic Curve (Performance of Preoperative Parameters in Discriminating the Risk of unfavorable outcome (MACE + cardiovascular hospitalization) 18 months after CABG).

Test Result Variable(s)	Area	Std. Error[a]	Asymptotic Sig.[b]	Asymptotic 95% Confidence Interval	
				Lower Bound	Upper Bound
**IVST**	**0.631**	**0.049**	0.010	0.535	0.727
NYHA III FC	0.596	0.052	0.057	0.494	0.699
Arrhythmia	0.579	0.052	0.117	0.477	0.682
Aspirin	0.441	0.053	0.241	0.337	0.544

The test result variable(s): IVS, NYHA III FK, Arrhitmia, Aspirin has at least one tie between the positive actual state group and the negative actual state group; a. Under the nonparametric assumption; IVST, interventricular septum thickness; NYHA, New York Heart Association; FC, functional class.
